# Trends in Prevalence of Hypertension and Hypertension Phenotypes Among Chinese Children and Adolescents Over Two Decades (1991–2015)

**DOI:** 10.3389/fcvm.2021.627741

**Published:** 2021-05-11

**Authors:** Xinxin Ye, Qian Yi, Jing Shao, Yan Zhang, Mingming Zha, Qingwen Yang, Wei Xia, Zhihong Ye, Peige Song

**Affiliations:** ^1^School of Public Health, Zhejiang University School of Medicine, Zhejiang University, Hangzhou, China; ^2^School of Nursing, Zhejiang University School of Medicine, Zhejiang University, Hangzhou, China; ^3^Faculty of Life Science and Medicine, Kings College London, London, United Kingdom; ^4^Medical School Southeast University, Nanjing, China; ^5^School of Nursing, Sun Yat-Sen University, Guangdong, China; ^6^Women's Hospital, Zhejiang University School of Medicine, Zhejiang University, Hangzhou, China

**Keywords:** hypertension, hypertension phenotypes, China, children, adolescents, trends

## Abstract

**Background:** Hypertension is a leading cause of cardiovascular-related morbidity and mortality. Elevated blood pressure (BP) in children is related to long-term adverse health effects. Until recently, few studies have reported the secular trend and associated factors of hypertension phenotypes in Chinese children and adolescents.

**Methods:** From the China Health and Nutrition Survey (CHNS) 1991–2015, a total of 15,143 records of children aged 7–17 years old were included. Following definitions of hypertension from the Chinese Child Blood Pressure References Collaborative Group, we estimated the prevalence of prehypertension, hypertension, stage 1 hypertension, stage 2 hypertension and its phenotypes (ISH, isolated systolic hypertension; IDH, isolated diastolic hypertension; SDH, combined systolic and diastolic hypertension). General estimation equation was used to analyze the trends in the prevalence of hypertension and hypertension phenotypes, and a multivariable logistic regression was constructed to explore the associated factors.

**Results:** During 1991–2015, increasing trends were revealed in BP and hypertension prevalence (*P* < 0.001) in Chinese children and adolescents. For ISH, IDH and SDH, the age-standardized prevalence increased dramatically from 0.9 to 2.2%, from 6.2 to 14.1%, and from 1.4 to 2.9%, respectively (all *P* < 0.001). Adolescents aged 13–17 years (OR = 1.76, 95% CI: 1.56–1.97, *P* < 0.001), general obesity (OR = 2.69, 95% CI: 2.10-3.44, *P* < 0.001) and central obesity (OR = 1.49, 95% CI: 1.21–1.83, *P* < 0.001) were positively associated with hypertension, whereas the South region (OR = 0.65, 95% CI: 0.58–0.73, *P* < 0.001) was a negative factor. Furthermore, body mass index (BMI) and general obesity were linked to the presence of ISH, IDH and SDH. Age, waist circumference (WC) and central obesity were additionally associated with ISH, and sex, age, urban/rural setting, North/South region, WC and central obesity were additionally associated with IDH.

**Conclusion:** BP levels and prevalence of hypertension and phenotypes increased dramatically in Chinese children and adolescents from 1991 to 2015. Regional discrepancy, demographic features, BMI, WC and overweight/obesity status were associated factors of hypertension among youths.

## Introduction

Hypertension, or elevated blood pressure (BP), is a highly prevalent chronic disease globally. Hypertension has been recognized as a primary modifiable contributor to cardiovascular and cerebrovascular diseases (1). In China, hypertension affects more than 270 million people, emerging as a major public health challenge (2–4). It was estimated that the direct costs caused by hypertension reached 210.3 billion Yuan in 2013, accounting for 6.61% of the total Chinese health-care expenditure (5). Hypertension is not a disorder that confines to adults, globally, millions of children were suffering from hypertension (6). Pathophysiologic and epidemiologic evidence has shown an association between childhood hypertension and long-term adverse health effects (7).

Unlike adulthood hypertension, whose definition has been upgraded to a universally diagnostic standard, the measurement of childhood hypertension is comparatively complicated (8). The cutoffs of childhood hypertension have been suggested as a systolic blood pressure (SBP) or a diastolic blood pressure (DBP) equal to or higher than 95th percentile by age, sex, and height (9). According to the fourth report from the National High Blood Pressure Education Program (NHBPEP) Working Group in the United States, BP in children and adolescents was suggested to be measured on at least three separate occasions at an interval of 2 weeks due to the apparent fluctuations (10). In 2017, the American Academy of Pediatrics (AAP) guidelines recommended the use of ambulatory BP monitoring in the diagnosis and management of hypertension in children (11).

China is the largest developing country across the world, where the emergence of hypertension has brought a heavy burden to the whole country (8, 10, 12, 13). A cohort study in China revealed that the hypertension prevalence was 17.00% in boys and 14.13% in girls aged 7–17 years (14), and it ranged from 5.2 to 7.8% in Italy among school-aged children (15, 16). Several previous studies have focused on the prevalence of hypertension, whereas studies on trends in different hypertension phenotypes were limited in China. Early identification of different hypertension phenotypes in youth is of significant importance in preventing cardiovascular events in adulthood (17). Compared with previous studies, we extended the study period to 1991–2015. We additionally assessed the prevalence of hypertension severity (stage one hypertension and stage two hypertension) and phenotypes (ISH, isolated systolic hypertension; IDH, isolated diastolic hypertension; SDH, systolic and diastolic hypertension) by age and sex, and explored the effects of demographic, geographic, anthropometric factors and obesity on childhood hypertension in China.

We herein hypothesized that the prevalence of hypertension and hypertension phenotypes in children increased gradually with years; the secular trends in childhood hypertension prevalence differed by age, sex, locations, and region; demographic, anthropometric, geographic factors, and general/central obesity were independently associated with increased BP and hypertension phenotypes in Chinese children and adolescents.

## Materials and Methods

### Study Design and Study Population

Details about the study design of CHNS are available elsewhere (18, 19). In brief, CHNS is a longitudinal health and nutrition survey in China, beginning in 1989 and having been continuously conducted every 2 or 4 years. So far, CHNS has been conducted for 10 rounds (1989, 1991, 1993, 1997, 2000, 2004, 2006, 2009, 2011, and 2015) and covered a set of large provinces across China (the list and locations of investigated provinces are shown in Supplementary Table 1 and Supplementary Figure 1). In each round, a multistage random-cluster sampling method was adopted to ensure a good representative of the general Chinese population. First, all counties and cities in each province were stratified into three groups (low-, middle- and high-income). Then, four counties (one low-, two middle-, and one high-income county) and two cities (usually the provincial capital and a lower-income city) were randomly selected. Third, one community and three rural villages within each selected county and two communities and two suburban villages within each selected city were randomly chosen. In each community or village, all members of 20 households were randomly selected (18, 19). A total of 15,143 records (7,423 subjects) aged 7–17 years old with complete data from 1991 to 2015 were included in this study, and the number of participants who participated twice or more was 4,865. The sample sizes of the nine rounds were 2,429 in 1991, 2,254 in 1993, 2,254 in 1997, 2,217 in 2000, 1,364 in 2004, 1,145 in 2006, 1,012 in 2009, 1,404 in 2011 and 1,064 in 2015 (the excluded and included records are shown in Table 1). CHNS was approved by the Institutional Review Committees of the University of North Carolina at Chapel Hill, the National Institute of Nutrition and Food Safety, the Chinese Center for Disease Control and Prevention, and the China-Japan Friendship Hospital, Ministry of Health. All respondents signed informed consent.

**Table 1:** Comparison of demographic characteristics between the excluded and included subjects in CHNS 1991–2015.

**Characteristic**	**1991–2015 combined**			**1991**			**1993**			**1997**			**2000**		
	**Excluded**	**Included**	***p***	**Excluded**	**Included**	***P***	**Excluded**	**Included**	***p***	**Excluded**	**Included**	***p***	**Excluded**	**Included**	***P***
	**3,460**	**15,143**	**value**	**508**	**2,429**	**value**	**527**	**2,254**	**value**	**622**	**2,254**	**value**	**660**	**2,217**	**value**
**AGE GROUP**															
7–12 years	1,526 (44.10%)	8,783 (58.00%)	<0.001	229 (45.08%)	1,335 (54.96%)	<0.001	246 (46.68%)	1,285 (57.01%)	<0.001	271 (43.57%)	1,358 (60.25%)	<0.001	207 (31.36%)	1,207 (54.44%)	<0.001
13–17 years	1,934 (55.90%)	6,360 (42.00%)		279 (54.92%)	1,094 (45.04%)		281 (53.32%)	969 (42.99%)		351 (56.43%)	896 (39.75%)		453 (68.64%)	1,010 (45.56%)	
**SEX**															
Male	1,842 (53.24%)	7,947 (52.48%)	0.421	277 (54.53%)	1,251 (51.50%)	0.215	256 (48.58%)	1,175 (52.13%)	0.142	333 (53.54%)	1,192 (52.88%)	0.773	342 (51.82%)	1,176 (53.04%)	0.58
Female	1,618 (46.76%)	7,196 (47.52%)		231 (45.47%)	1,178 (48.50%)		271 (51.42%)	1,079 (47.87%)		289 (46.46%)	1,062 (47.12%)		318 (48.18%)	1,041 (46.96%)	
**LOCATION**															
Urban	866 (25.03%)	4,347 (28.71%)	<0.001	112 (22.05%)	620 (25.52%)	0.099	110 (20.87%)	566 (25.11%)	0.041	142 (23.00%)	659 (29.24%)	0.002	161 (24.39%)	609 (27.47%)	0.117
Rural	2,594 (74.97%)	10,796 (71.29%)		396 (77.95%)	1,809 (74.48%)		417 (79.13%)	1,688 (74.89%)		480 (77.00%)	1,595 (70.76%)		499 (75.61%)	1,608 (72.53%)	
**REGION**															
North	1,434 (41.45%)	5,286 (34.91%)	<0.001	226 (44.49%)	728 (29.97%)	<0.001	228 (43.26%)	698 (30.97%)	<0.001	221 (35.53%)	776 (34.43%)	0.609	266 (40.3%)	964 (43.48%)	0.147
South	2,026 (58.55%)	9,857 (65.09%)		282 (55.51%)	1,701 (70.03%)		299 (56.74%)	1556 (69.03%)		401 (64.47%)	1,478 (65.57%)		394 (59.70%)	1,253 (56.52%)	
**BMI (kg/m**^**2**^**)**	17.42 ± 4.63	17.69 ± 3.25	0.040	15.4 ± 1.86	17.38 ± 2.78	<0.001	15.66 ± 1.94	17.28 ± 2.68	<0.001	15.66 ± 2.05	17.43 ± 2.78	<0.001	16.46 ± 2.41	17.68 ± 2.85	0.004
**WC (cm)**	59.01 ± 12.85	62.87 ± 9.9	<0.001	NA	NA		56.48 ± 6.8	62.39 ± 8.5	<0.001	55.98 ± 6.45	61.39 ± 8.17	<0.001	58.42 ± 7.12	62.8 ± 8.38	<0.001
**GENERAL OBESITY**															
Normal	568 (85.80%)	13,228 (88.06%)	0.08	83 (94.32%)	2,222 (92.20%)	0.47	86 (93.48%)	2,046 (91.96%)	0.60	101 (93.52%)	2,042 (91.12%)		44 (95.65%)	1,983 (90.05%)	0.21
Overweight/ Obesity	94 (14.20%)	1,794 (11.94%)		5 (5.68%)	188 (7.80%)		6 (6.52%)	179 (8.04%)		7 (6.48%)	199 (8.88%)	0.39	2 (4.35%)	219 (9.95%)	
**CENTRAL OBESITY**															
Normal	567 (91.60%)	10,571 (91.68%)	0.94	NA	NA		66 (95.65%)	1,217 (96.36%)	0.76	128 (96.24%)	2,078 (96.25%)	1.00	72 (94.74%)	2,068 (94.65%)	0.97
Central Obesity	52 (8.40%)	959 (8.32%)		NA	NA		3 (4.35%)	46 (3.64%)		5 (3.76%)	81 (3.75%)		4 (5.26%)	117 (5.35%)	
**AGE GROUP**															
7–12 years	158 (55.83%)	646 (47.36%)	0.009	74 (48.37%)	667 (58.25%)	0.02	63 (49.61%)	618 (61.07%)	0.013	42 (52.5%)	874 (62.25%)	0.081	236 (47.2%)	793 (74.53%)	<0.001
13–17 years	125 (44.17%)	718 (52.64%)		79 (51.63%)	478 (41.75%)		64 (50.39%)	394 (38.93%)		38 (47.5%)	530 (37.75%)		264 (52.8%)	271 (25.47%)	
**Sex**															
Male	154 (54.42%)	720 (52.79%)	0.617	85 (55.56%)	609 (53.19%)	0.581	72 (56.69%)	562 (55.53%)	0.804	41 (51.52%)	713 (50.78%)	0.935	282 (56.4%)	549 (51.60%)	0.047
Female	129 (45.58%)	644 (47.21%)		68 (44.44%)	536 (46.81%)		55 (43.31%)	450 (44.47%)		39 (48.75%)	691 (49.22%)		218 (43.6%)	515 (48.40%)	
**LOCATION**															
Urban	59 (20.85%)	408 (29.91%)	0.002	41 (26.80%)	343 (29.96%)	0.421	35 (27.56%)	274 (27.08%)	0.908	34 (42.50%)	530 (37.75%)	0.395	172 (34.40%)	338 (31.77%)	0.3
Rural	224 (79.15%)	956 (70.09%)		112 (73.20%)	802 (70.04%)		92 (72.44%)	738 (72.92%)		46 (57.50%)	874 (62.25%)		328 (66.00%)	726 (68.23%)	
**REGION**															
North	138 (48.76%)	556 (40.76%)	0.013	77 (50.33%)	445 (38.86%)	0.007	40 (31.50%)	375 (37.06%)	0.22	35 (43.75%)	442 (31.48%)	0.022	203 (40.60%)	302 (28.38%)	<0.001
South	145 (51.24%)	808 (59.24%)		76 (49.67%)	700 (61.14%)		87 (68.50%)	637 (62.94%)		45 (56.25%)	962 (68.52%)		297 (59.40%)	762 (71.62%)	
**BMI, kg/m**^**2**^	16.54 ± 2.71	18.1 ± 2.92	<0.001	16.65 ± 2.93	17.77 ± 3.09	0.109	16.96 ± 4.28	17.79 ± 3.35	0.4368	15.57 ± 2.14	18.35 ± 3.8	0.0208	21.12 ± 6.52	18.28 ± 5.6	<0.001
**WC, cm**	57.73 ± 8.53	64.1 ± 9.47	<0.001	57.55 ± 7.63	62.52 ± 10.01	0.005	67.49 ± 7.36	63.07 ± 10.01	0.1273	55.57 ± 7.21	64.49 ± 11.96	0.0683	63.11 ± 20.26	63.03 ± 13.71	0.9487
**GENERAL OBESITY**															
Normal	87 (87.88%)	1,181 (87.35%)	0.88	14 (70.00%)	979 (87.49%)	0.02	9 (90.00%)	845 (83.83%)	0.60	10 (100.00%)	1,111 (79.13%)	0.11	134 (70.90%)	819 (77.19%)	0.06
Overweight/ Obesity	12 (12.12%)	171 (12.65%)		6 (30.00%)	140 (12.51%)		1 (10.00%)	163 (16.17%)		0 (0.00%)	293 (20.87%)		55 (29.10%)	242 (22.81%)	
**CENTRAL OBESITY**															
Normal	105 (94.59%)	1,243 (92.21%)	0.36	33 (100.00%)	1,047 (93.32%)	0.13	10 (83.33%)	898 (89.62%)	0.48	6 (100.00%)	1,168 (83.31%)	0.27	147 (82.12%)	852 (81.22%)	0.77
Central Obesity	6 (5.41%)	105 (7.79%)		0 (0.00%)	75 (6.68%)		2 (16.67%)	104 (10.38%)		0 (0.00%)	234 (16.69%)		32 (17.88%)	197 (18.78%)	

*Data are presented as means ± standard deviations or n (%); BMI, Body Mass Index; WC, Waist Circumference*.

### Data Collection

Data on demographics (age and sex) and geographic location (urban/rural and North/South region) were collected by trained interviewers using a structured questionnaire. BP, weight, height, waist circumference (WC) and hip circumference (HC) were measured following standardized protocols from the World Health Organization (20). BP was calculated as the mean of three measurements with an interval of 3–5 min by standard mercury sphygmomanometer (11, 21, 22). Weight was measured without coats to the nearest 0.1 kg on a calibrated beam scale, and height was measured without shoes to the nearest 0.1 cm using a portable stadiometer. WC was taken at the end of expiratory period at the midpoint of the line between the lower rib and the upper iliac crest (23, 24). HC was measured at the level of maximal gluteal protrusion with a non-elastic tape. Body mass index (BMI) was calculated as weight divided by height squared (kg/m^2^), waist to height ratio (WHtR) as WC divided by height, and waist to hip ratio (WHR) as WC divided by HC.

### Definitions

#### Hypertension and Phenotypes

Conforming to the standard of the Chinese Child Blood Pressure References Collaborative Group, the definitions of childhood hypertension and phenotypes are listed in Table 2 (25).

**Table 2:** Classification of blood pressure and definitions of hypertension and hypertension phenotypes in children and adolescents.

**Category**	**Definition**
Normal	SBP with DBP less the 90th percentile (for age, sex and height)
Prehypertension	An SBP and/or DBP between the 90th and 95th percentile (for age, sex and height) or ≥120/80 mm Hg
Hypertension	An SBP and/or DBP≥95th percentile (for age, sex and height) on ≥3 separate occasions
Stage 1	An SBP and/or DBP between 95th and 99th percentile plus 5 mmHg (for age, sex and height) on ≥3 separate occasions
Stage 2	An SBP and/or DBP above 99th percentile plus 5 mmHg (for age, sex and height) on ≥3 separate occasions
ISH	An SBP≥95th percentile (for age, sex, and height) but a DB*P < *95th percentile (for age, sex, and height) on ≥3 separate occasions
IDH	A DBP≥95th percentile (for age, sex, and height) but an SB*P < * 95th percentile (for age, sex, and height) on ≥3 separate occasions
SDH	An SBP and DBP≥95th percentile (for age, sex, and height) on ≥3 separate occasions

^*^ SBP, systolic blood pressure; DBP, diastolic blood pressure; IDH, isolated diastolic hypertension; ISH, isolated systolic hypertension; SDH, systolic-diastolic hypertension.

#### Demographics, Geographic Locations, General, and Central Obesity

Following the criteria of the 2017 AAP guidelines, we divided all participants into two age groups of 7–12 and 13–17 years (11). Residence was classified into South China (Shanghai, Jiangsu, Hubei, Hunan, Guangxi, Guizhou, and Chongqing in CHNS) and North China (Beijing, Liaoning, Heilongjiang, Shandong, and Henan in CHNS). Using the Working Group on Obesity in China criteria, a BMI between 85th and 95th percentile of sex and age group was defined as overweight, and ≥95th percentile as obesity. Central obesity was defined as a WC> 90th percentile in each sex and age group, or a WHtR ≥ 0.5, or a WHR ≥ 0.9 in boys or ≥0.85 in girls (26).

### Statistical Analysis

In descriptive analyses, continuous variables were reported as mean and standard deviation (SD) and categorical data were presented as percentage (%) with 95% confidence interval (CI). Using the Chi-square test, we compared the age and sex distributions between the excluded and included groups. Given that the same participant might be included in different survey rounds, generalized estimating equation (GEE) was adopted (27, 28). Individual calendar year was selected as a single continuous variable and age were adjusted for in GEE models to examine the trends in the prevalence of childhood hypertension and phenotypes. Subgroup trend analyses from 1991 to 2015 were conducted by strata of age and sex. To estimate the associated factors of childhood hypertension and phenotypes, multivariable logistic regression was carried out. All statistical analyses were performed using Stata statistical software (version 14.0; Stata Corporation, College Station, TX, USA), and a *P* < 0.05 was considered as statistically significant in two-sided tests.

## Results

### Characteristics of the Participants

A total of 18,603 records of children and adolescents were available from CHNS 1991 to 2015, of which 15,143 were with BP measurements. The basic characteristics (demographics, anthropometry and geography) are shown in Table 3. The flow chart of this study is shown in Supplementary Figure 2.

**Table 3:** Characteristics of included subjects in CHNS 1991–2015.

**Characteristic**	**1991–2015 combined (15,143)**	**1991 (2,429)**	**1993 (2,254)**	**1997 (2,254)**	**2000 (2,217)**	**2004 (1,364)**	**2006 (1,145)**	**2009 1,012**	**2011 (1,404)**	**2015 (1,064)**
**AGE GROUP**										
7–12 years	8,783 (58.00)	1,335 (54.96)	1,285 (57.01)	1,358 (60.25)	1,207 (54.44)	646 (47.36)	667 (58.25)	618 (61.07)	874 (62.25)	793 (74.53)
13–17 years	6,360 (42.00)	1,094 (45.04)	969 (42.99)	896 (39.75)	1,010 (45.56)	718 (52.64)	478 (41.75)	394 (38.93)	530 (37.75)	271 (25.47)
**SEX**										
Male	7,947 (52.48)	1,251 (51.50)	1,175 (52.13)	1,192 (52.88)	1,176 (53.04)	720 (52.79)	609 (53.19)	562 (55.53)	713 (50.78)	549 (51.60)
Female	7,196 (47.52)	1,178 (48.50)	1,079 (47.87)	1,062 (47.12)	1,041 (46.96)	644 (47.21)	536 (46.81)	450 (44.47)	691 (49.22)	515 (48.40)
**ANTHROPOMETRIC MEASURES**										
Height, cm	144.65 ± 0.26	142.37 ± 0.64	142.11 ± 0.68	142.62 ± 0.65	146.41 ± 0.63	149.05 ± 0.86	146.04 ± 1.00	146.44 ± 0.99	146.47 ± 0.88	144.55 ± 0.97
Weight, kg	38.18 ± 0.21	36.39 ± 0.49	36.05 ± 0.50	36.55 ± 0.50	38.89 ± 0.50	41.33 ± 0.69	39.12 ± 0.77	39.29 ± 0.82	40.67 ± 0.77	39.35 ± 0.96
**BMI, kg/m**^**2**^	17.69 ± 0.05	17.38 ± 0.11	17.28 ± 0.12	17.43 ± 0.11	17.68 ± 0.11	18.10 ± 0.15	17.77 ± 0.18	17.79 ± 0.20	18.35 ± 0.20	18.28 ± 0.34
**WC, cm**	62.87 ± 0.19	NA	62.39 ± 0.47	61.39 ± 0.35	62.80 ± 0.35	64.10 ± 0.50	62.52 ± 0.58	63.07 ± 0.62	64.49 ± 0.63	63.03 ± 0.83
**BLOOD PRESSURE MEASURES**										
SBP, mmHg	98.44 ± 0.20	96.18 ± 0.53	96.34 ± 0.54	97.18 ± 0.53	99.49 ± 0.54	101.53 ± 0.70	98.00 ± 0.73	99.83 ± 0.78	100.12 ± 0.65	101.54 ± 0.76
DBP, mmHg	64.57 ± 0.15	62.61 ± 0.39	63.25 ± 0.41	63.67 ± 0.40	65.15 ± 0.39	66.66 ± 0.51	64.74 ± 0.52	66.63 ± 0.57	65.36 ± 0.47	66.65 ± 0.55
**SETTING**										
Urban	4,347 (28.71)	620 (25.52)	566 (25.11)	659 (29.24)	609 (27.47)	408 (29.91)	343 (29.96)	274 (27.08)	530 (37.75)	338 (31.77)
Rural	10,796 (71.29)	1,809 (74.48)	1,688 (74.89)	1,595 (70.76)	1,608 (72.53)	956 (70.09)	802 (70.04)	738 (72.92)	874 (62.25)	726 (68.23)
**REGION**										
North	5,286 (34.91)	728 (29.97)	698 (30.97)	776 (34.43)	964 (43.48)	556 (40.76)	445 (38.86)	375 (37.06)	442 (31.48)	302 (28.38)
South	9,857 (65.09)	1,701 (70.03)	1,556 (69.03)	1,478 (65.57)	1,253 (56.52)	808 (59.24)	700 (61.14)	637 (62.94)	962 (68.52)	762 (71.62)

^*^Data are expressed as n (%) or mean ± SD; BMI, body mass index; WC, Waist circumference; SBP, systolic blood pressure; DBP, diastolic blood pressure. NA, Not available.

### Trends in SBP, DBP, and Prevalence of Childhood Hypertension and Phenotypes

Table 4 shows the changes in age-standardized mean SBP and DBP by strata of age and sex. From 1991 to 2015, SBP and DBP values increased significantly from 96.18 mmHg (95% CI: 95.65–96.71) to 101.54 mmHg (95% CI: 100.78–102.30), and from 62.61 mmHg (95% CI: 62.22–63.01) to 66.65 mmHg (95% CI: 66.10–67.20), respectively (both *P*-values for age-adjusted trend< 0.001). Mean SBP and DBP values increased across age and sex groups during the same time period, with an average annual increase (AAI) ranging from 0.12 mmHg to 0.37 mmHg and an average relative increase (ARI) from 0.19 to 0.46%.

**Table 4:** Mean SBP and DBP in Chinese children and adolescents, CHNS 1991–2015.

**Variable**	**1991 (*n =* 2,429)**	**1993 (*n =* 2,254)**	**1997 (*n =* 2,254)**	**2000 (*n =* 2,217)**	**2004 (*n =* 1,364)**	**2006 (*n =* 1,145)**	**2009 (*n =* 1,012)**	**2011 (*n =* 1,404)**	**2015 (*n =* 10,64)**	**AAI (%)**	**ARI (%)**	**P for age-adjusted trend**
**SBP (mmHg), mean (95% CI)**												
Overall	96.18	96.34	97.18	99.49	101.53	98.00	99.83	100.12	101.54	0.22	0.23	<0.001
	(95.65–96.71)	(95.8–96.88)	(96.65–97.72)	(98.95–100.02)	(100.83–102.24)	(97.27–98.73)	(99.05–100.62)	(99.47–100.77)	(100.78–102.3)			
**AGE GROUP**												
7–12 years	90.47	91.59	92.65	95.14	95.27	92.98	96.54	96.24	99.23	0.37	0.40	<0.001
	(89.88–91.06)	(90.93–92.25)	(92.02–93.27)	(94.48–95.8)	(94.32–96.21)	(92.12–93.85)	(95.54–97.55)	(95.47–97)	(98.37–100.09)			
13–17 years	103.15	102.64	104.05	104.68	107.17	105.00	104.99	106.52	108.29	0.21	0.21	<0.001
	(102.4–103.9)	(101.91–103.37)	(103.29–104.82)	(103.92–105.44)	(106.34–108.01)	(104.02–105.97)	(103.93–106.06)	(105.57–107.46)	(106.94–109.65)			
**SEX**												
Male	96.51	96.67	97.19	100.32	102.48	98.76	100.20	101.24	102.90	0.27	0.28	<0.001
	(95.76–97.26)	(95.88–97.45)	(96.45–97.93)	(99.56–101.09)	(101.47–103.48)	(97.71–99.81)	(99.11–101.29)	(100.29–102.19)	(101.81–103.99)			
Female	95.83	95.99	97.17	98.54	100.48	97.13	99.37	98.96	100.09	0.18	0.19	<0.001
	(95.08–96.58)	(95.26–96.72)	(96.39–97.95)	(97.8–99.29)	(99.52–101.45)	(96.12–98.15)	(98.25–100.5)	(98.09–99.84)	(99.03–101.14)			
**DBP (mmHg), mean (95% CI)**												
Overall	62.61	63.25	63.67	65.15	66.66	64.74	66.63	65.36	66.65	0.17	0.27	<0.001
	(62.22–63.01)	(62.84–63.66)	(63.27–64.08)	(64.76–65.53)	(66.15–67.18)	(64.22–65.26)	(66.06–67.2)	(64.89–65.84)	(66.1–67.2)			
**AGE GROUP**												
7–12 years	58.99	60.23	60.77	62.49	63.09	61.52	64.77	62.95	65.49	0.27	0.46	<0.001
	(58.5–59.47)	(59.7–60.76)	(60.28–61.27)	(61.99–62.99)	(62.35–63.83)	(60.91–62.12)	(64.04–65.5)	(62.38–63.52)	(64.86–66.11)			
13–17 years	67.04	67.26	68.07	68.33	69.88	69.24	69.55	98.96	100.09	0.12	0.19	<0.001
	(66.5–67.58)	(66.7–67.82)	(67.49–68.65)	(67.78–68.87)	(69.26–70.5)	(68.48–70)	(68.71–70.38)	(68.64–70.07)	(68.97–71.09)			
**SEX**												
Male	62.66	63.43	63.58	65.55	67.13	65.14	66.67	66.36	67.46	0.20	0.32	<0.001
	(62.1–63.21)	(62.84–64.03)	(63.04–64.12)	(65.01–66.09)	(66.41–67.84)	(64.39–65.89)	(65.9–67.44)	(65.67–67.05)	(66.66–68.26)			
Female	62.57	63.06	63.78	64.69	66.15	64.29	66.58	64.34	65.78	0.13	0.21	<0.001
	(62.01–63.13)	(62.48–63.63)	(63.17–64.38)	(64.14–65.24)	(65.41–66.88)	(63.58–65.01)	(65.73–67.43)	(63.69–64.98)	(65.03–66.52)			

*AAI, Average annual increase, AAI was computed by dividing the difference of blood pressure between 1991 and 2015 by the number of years covered; ARI = Average relative increase, ARI was calculated by dividing the average annual change by the baseline mean blood pressure in 1991; trends in mean blood pressure from 1991 to 2015 were assessed by the generalized-estimating-equation method and adjusted by age and sex*.

Table 5 presents the trends of age-standardized prevalence of childhood hypertension and phenotypes. For prehypertension, the age-standardized prevalence significantly increased from 7.0% (95% CI: 6.0–8.1) in 1991 to 13.00% (95% CI: 11.1–15.1) in 2015. Prehypertension was consistently more prevalent in boys (than in girls) and in teenagers aged 13–17 years (than in those aged 7–12 years). For childhood hypertension, the overall age-standardized prevalence rose from 8.5% (95% CI: 7.4–9.7) to 19.2% (95% CI: 16.9–21.7) across the same study time period, yielding a relative increasing rate of 5.3% (*P* < 0.001). The prevalence of childhood hypertension was generally higher in participants aged 13–17 years (than in the younger groups). Besides, the age-standardized prevalence of prehypertension and hypertension increased in all age and sex subgroups (*P* < 0.001) and the prevalence of hypertension in children aged 7–12 years increased the most during the survey rounds (increased by 12.7%).

**Table 5:** Prevalence of hypertension and phenotypes in Chinese children and adolescents, CHNS 1991–2015.

**Variable**	**1991 (*n =* 2,429)**	**1993 (*n =* 2,254)**	**1997 (*n =* 2254)**	**2000 (*n =* 2217)**	**2004 (*n =* 1,364)**	**2006 (*n =* 1,145)**	**2009 (*n =* 1,012)**	**2011 (*n =* 1404)**	**2015 (*n =* 1,064)**	**AAI (%)**	**ARI (%)**	***P* for age-adjusted trend**
**Prehypertension (%, 95% CI)**												
Overall	7.0 (6.0–8.1)	7.5 (6.4–8.6)	8.5 (7.4–9.7)	9.2 (8.0–10.4)	9.9 (8.4–11.6)	9.2 (7.6–11.0)	11.3 (9.5–13.4)	9.9 (8.4–11.6)	13.0 (11.1–15.1)	0.3	3.6	<0.001
**AGE GROUP**												
7–12 years	5.7 (4.6–7.1)	6.1 (5–7.6)	6.5 (5.3–7.9)	6.5 (5.3–8.1)	7.1 (5.4–9.1)	6.1 (4.6–8.2)	9.5 (7.5–12.1)	6.3 (4.9–8.1)	12.7 (10.6–15.2)	0.3	5.2	<0.001
13–17 years	8.6 (7.1–10.4)	9.2 (7.5–11.2)	11.6 (9.7–13.9)	12.3 (10.4–14.5)	12.4 (10.2–15.0)	13.4 (10.6–16.8)	14.0 (10.9–17.8)	15.8 (13.0–19.2)	13.7 (10.0–18.3)	0.2	2.5	<0.001
**SEX**												
Male	6.6 (5.3–8.1)	7.5 (6.1–9.1)	8.7 (7.2–10.5)	9.5 (8.0–11.3)	11.7 (9.5–14.2)	9.5 (7.4–12.1)	11.9 (9.5–14.9)	11.8 (9.6–14.4)	13.3 (10.7–16.4)	0.3	4.3	<0.001
Female	7.5 (6.1–9.1)	7.4 (6.0–9.1)	8.3 (6.8–10.1)	8.7 (7.2–10.6)	7.9 (6.1–10.3)	8.8 (6.6–11.5)	10.4 (7.9–13.6)	8.0 (6.2–10.2)	12.6 (10.0–15.8)	0.2	2.9	0.001
**Hypertension (%, 95% CI)**												
Overall	8.5 (7.4–9.7)	9.6 (8.5–10.9)	11.1 (9.9–12.5)	12.4 (11.1–13.8)	16.9 (15.0–18.9)	9.9 (8.3–11.7)	15.9 (13.8–18.3)	12.6 (11.0–14.4)	19.2 (16.9–21.7)	0.5	5.3	<0.001
**AGE GROUP**												
7–12 years	4.5 (3.5–5.7)	8.0 (6.7–9.6)	8.1 (6.8–9.7)	9.9 (8.3–11.7)	13.2 (10.8–16)	6.3 (4.7–8.4)	16.3 (13.6–19.5)	11.3 (9.4–13.6)	18.2 (15.6–21.0)	0.6	12.7	<0.001
13–17 years	13.3 (11.5–15.5)	11.8 (9.9–14.0)	15.7 (13.5–18.3)	15.3 (13.3–17.7)	20.2 (17.4–23.3)	14.9 (11.9–18.3)	15.2 (12.0–19.1)	14.7 (11.9–18.0)	22.1 (17.6–27.5)	0.4	2.8	<0.001
**SEX**												
Male	9.0 (7.5–10.7)	10.1 (8.5–12)	10.1 (8.5–11.9)	13.2 (11.4–15.2)	16.4 (13.9–19.3)	10.2 (8.8–12.8)	14.4 (11.7–17.6)	14.2 (11.8–16.9)	21.5 (18.3–25.1)	0.5	5.8	<0.001
Female	8.0 (6.6–9.7)	9.1 (7.5–11.0)	12.3 (10.5–14.5)	11.4 (9.6–13.5)	17.4 (14.7–20.5)	9.5 (7.3–12.3)	17.8 (14.5–21.6)	11.0 (8.9–13.6)	16.7 (13.7–20.2)	0.4	4.6	<0.001
**Stage 1 hypertension (%, 95% CI)**												
Overall	8.0 (7.0–9.2)	9.1 (8.0–10.4)	10.6 (9.4–11.9)	11.7 (10.5–13.1)	16.0 (14.1–18)	9.7 (8.1–11.5)	14.6 (12.6–16.9)	12.3 (10.6–14.1)	17.7 (15.5–20.1)	0.4	5.0	<0.001
**AGE GROUP**												
7–12 years	4.3 (3.3–5.5)	7.5 (6.2–9.1)	7.7 (6.4–9.3)	9.3 (7.8–11.1)	12.1 (9.8–14.8)	6.3 (4.7–8.4)	14.7 (12.1–17.7)	11.0 (9.1–13.2)	16.4 (14–19.1)	0.5	11.8	<0.001
13–17 years	12.6 (10.8–14.7)	11.1 (9.3–13.3)	14.8 (12.7–17.3)	14.7 (12.6–17.0)	19.5 (16.8–22.6)	14.4 (11.6–17.9)	14.5 (11.3–18.3)	14.3 (11.6–17.6)	21.4 (16.9–26.7)	0.4	2.9	<0.001
**SEX**												
Male	8.7 (7.3–10.4)	9.4 (7.9–11.3)	9.8 (8.3–11.6)	12.4 (10.6–14.4)	15.6 (13.1–18.4)	9.9 (7.7–12.5)	13.2 (10.6–16.2)	13.7 (11.4–16.5)	20.4 (17.2–24.0)	0.5	5.6	<0.001
Female	7.3 (5.9–8.9)	8.7 (7.2–10.5)	11.4 (9.6–13.4)	11.0 (9.2–13.0)	16.5 (13.8–19.5)	9.5 (7.3–12.3)	16.4 (13.3–20.2)	10.7 (8.6–13.2)	14.8 (11.9–18.1)	0.3	4.3	<0.001
**Stage 2 hypertension (%, 95% CI)**												
Overall	0.5 (0.3–0.8)	0.5 (0.3–0.9)	0.6 (0.3–1.0)	0.6 (0.4–1.1)	0.9 (0.5–1.5)	0.2 (0.0–0.7)	1.3 (0.7–2.2)	0.4 (0.1–0.9)	1.5 (0.9–2.4)	0.0	9.7	0.012
**AGE GROUP**												
7–12 years	0.2 (0.1–0.7)	0.5 (0.2–1.0)	0.4 (0.2–0.9)	0.6 (0.3–1.2)	1.1 (0.5–2.3)	0.0 (0.0–0.0)	1.6 (0.9–3.0)	0.3 (0.1–1.1)	1.8 (1.0–3.0)	0.06	28.6	<0.001
13–17 years	0.7 (0.4–1.5)	0.6 (0.3–1.4)	0.9 (0.4–1.8)	0.7 (0.3–1.4)	0.7 (0.3–1.7)	0.4 (0.1–1.7)	0.8 (0.2–2.3)	0.4 (0.1–1.5)	0.7 (0.2–2.9)	0.0	0.0	0.589
**SEX**												
Male	0.2 (0.1–0.7)	0.7 (0.3–1.4)	0.3 (0.1–0.8)	0.8 (0.4–1.5)	0.8 (0.4–1.8)	0.3 (0.1–1.3)	1.2 (0.6–2.6)	0.4 (0.1–1.3)	1.1 (0.5–2.4)	0.0	14.8	0.045
Female	0.7 (0.3–1.4)	0.4 (0.1–1.0)	0.9 (0.5–1.7)	0.5 (0.2–1.1)	0.9 (0.4–2.1)	0.0 (0.0–0.0)	1.3 (0.6–2.9)	0.3 (0.1–1.2)	1.9 (1.0–3.6)	0.1	7.8	0.110
**ISH (%, 95% CI)**												
Overall	0.9 (0.6–1.4)	0.8 (0.5–1.3)	1.6 (1.1–2.2)	1.4 (0.9–1.9)	2.1 (1.5–3.0)	1.1 (0.7–1.9)	1.6 (1.0–2.6)	1.1 (0.7–1.9)	2.2 (1.4–3.2)	0.1	5.4	0.001
**AGE GROUP**												
7–12 years	1 (0.6–1.8)	0.8 (0.4–1.5)	1.2 (0.7–2.0)	1.3 (0.8–2.1)	1.7 (0.9–2.9)	1.3 (0.7–2.6)	2.0 (1.1–3.5)	1.5 (0.9–2.8)	1.5 (0.7–2.9)	0.02	1.7	<0.001
13–17 years	0.8 (0.5–1.6)	0.8 (0.4–1.6)	2.0 (1.3–3.0)	1.4 (0.9–2.4)	2.6 (1.6–4.2)	0.9 (0.4–2.2)	1.1 (0.5–2.6)	0.7 (0.3–1.7)	2.9 (1.8–4.8)	0.1	10.1	0.932
**SEX**												
Male	0.3 (0.1–0.8)	0.5 (0.3–1.1)	1.0 (0.6–1.7)	1.3 (0.8–2.2)	1.1 (0.5–2.3)	1.2 (0.6–2.4)	1.9 (1.1–3.4)	1.3 (0.7–2.3)	2.0 (1.2–3.3)	0.1	23.9	0.027
Female	1.7 (1.1–2.7)	1.1 (0.6–2.0)	2.3 (1.5–3.6)	1.4 (0.8–2.3)	3.1 (2.0–4.6)	1.0 (0.4–2.5)	1.0 (0.4–2.7)	0.9 (0.4–2.2)	2.6 (1.2–5.3)	0.0	2.0	0.021
**IDH (%, 95% CI)**												
Overall	6.2 (5.3–7.2)	7.5 (6.5–8.7)	7.5 (6.5–8.7)	8.9 (7.8–10.1)	13.0 (11.3–14.9)	7.2 (5.8–8.8)	12.1 (10.2–14.2)	9.2 (7.8–10.8)	14.1 (12.1–16.3)	0.3	5.4	<0.001
**AGE GROUP**												
7–12 years	3.6 (2.7–4.7)	6.1 (4.9–7.5)	4.9 (3.9–6.2)	6.1 (4.9–7.6)	10.5 (8.4–13.1)	3.1 (2.1–4.8)	11.7 (9.3–14.4)	7.8 (6.2–9.8)	12.6 (10.5–15.1)	0.4	10.5	<0.001
13–17 years	9.3 (7.7–11.2)	9.5 (7.8–11.5)	11.4 (9.5–13.6)	12.2 (10.3–14.3)	15.2 (12.7–18)	12.8 (10.1–16.1)	12.7 (9.7–16.4)	11.5 (9.1–14.5)	18.5 (14.3–23.5)	0.4	4.1	<0.001
**SEX**												
Male	6.6 (5.4–8.2)	7.8 (6.4–9.5)	7.6 (6.3–9.3)	9.7 (8.1–11.5)	12.8 (10.5–15.4)	7.4 (5.6–9.8)	10.5 (8.2–13.3)	10.2 (8.2–12.7)	16.4 (13.5–19.7)	0.4	6.1	<0.001
Female	5.7 (4.5–7.2)	7.2 (5.8–8.9)	7.3 (5.9–9.1)	8.0 (6.5–9.8)	13.2 (10.8–16.0)	6.9 (5.0–9.4)	14.0 (11.1–17.5)	8.1 (6.3–10.4)	11.7 (9.1–14.7)	0.3	4.4	<0.001
**SDH (%, 95% CI)**												
Overall	1.4 (1.4–1.9)	1.3 (0.9–1.8)	2.1 (1.6–2.8)	2.1 (1.6–2.8)	1.8 (1.2–2.6)	1.6 (1.0–2.5)	2.3 (1.5–3.4)	2.3 (1.6–3.2)	2.9 (2.1–4.1)	0.1	4.8	0.001
**AGE GROUP**												
7–12 years	0.6 (0.3–1.2)	1.4 (0.9–2.2)	2.1 (1.5–3.1)	2.4 (1.7–3.4)	1.5 (0.8–2.9)	1.9 (1.1–3.3)	2.8 (1.7–4.4)	2.3 (1.5–3.5)	3.5 (2.4–5.1)	0.12	20.4	<0.001
13–17 years	2.3 (1.5–3.4)	1.1 (0.6–2.0)	2.0 (1.3–3.2)	1.8 (1.1–2.8)	1.9 (1.2–3.3)	1.0 (0.4–2.5)	1.5 (0.7–3.4)	2.3 (1.3–3.9)	1.1 (0.4–3.4)	−0.1	−2.2	0.632
**SEX**												
Male	1.3 (0.8–2.1)	1.5 (1.0–2.4)	1.3 (0.8–2.1)	2.2 (1.5–3.2)	1.9 (1.2–3.3)	1.5 (0.8–2.8)	2.0 (1.1–3.5)	2.4 (1.5–3.8)	3.6 (2.4–5.6)	0.1	7.7	0.002
Female	1.4 (0.9–2.3)	1.0 (0.6–1.8)	3.0 (2.1–4.2)	2.0 (1.3–3.1)	1.6 (0.8–2.9)	1.7 (0.9–3.2)	2.7 (1.5–4.6)	2.2 (1.3–3.6)	2.1 (1.2–3.8)	0.0	2.0	0.146

*AAI, Average annual increase, AAI was computed by dividing the difference of prevalence between 1991 and 2015 by the number of years covered; ARI=Average relative increase, ARI was calculated by dividing the average annual change by the baseline prevalence in 1991; trends in prevalence from 1991 to 2015 were assessed by the generalized–estimating-equation method and adjusted by age and sex*.

For stage 1 and stage 2 hypertension, the age-standardized prevalence increased from 8.0% (95% CI: 7.0–9.2) to 17.7% (95% CI: 15.5–20.1) and from 0.5% (95% CI: 0.3–0.8) to 1.5% (95% CI: 0.9–2.4), respectively. The prevalence of stage 1 hypertension was generally higher than that of stage 2 hypertension. Within this time frame, the ARI rates of both stage 1 and stage 2 hypertension were greater in 7–12 years old children (increased by 11.8% and 28.6%) than in older groups (increased by 2.9% and 0.0%). No statistically significant prevalence trend in stage 2 hypertension was observed among people aged 13–17 years and in girls (*P* = 0.589 and 0.110, separately).

**Figure 1:**
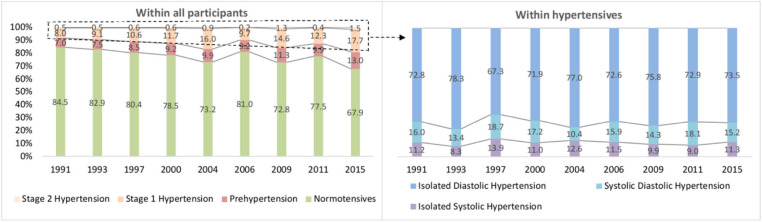
The proportions of childhood hypertension and phenotypes, CHNS 1991–2015.

IDH accounted for the largest share of hypertension phenotypes (Figure 1). For ISH, the age-standardized prevalence increased significantly from 0.9% (95% CI: 0.6–1.4) to 2.2% (95% CI: 1.4–3.2) from CHNS 1991 to CHNS 2015. Upward trends were observed in all subgroups (males, females and 7–12 years), except in people aged 13–17 (*P* = 0.932). For IDH, the age-standardized prevalence increased from 6.2% to 14.1% (boys from 6.6 to 16.4%, girls from 5.7 to 11.7%) over 25 years. The prevalence of IDH in two age groups (7–12 and 13–17 years) increased by 10.5% and 4.1% (both age-adjusted *P* < 0.001). The age-standardized prevalence of SDH increased from 1.4% (95% CI: 1.4–1.9) in 1991 to 2.9% (95% CI: 2.1–4.1) in 2015. An increasing trend of SDH prevalence was revealed in the age group of 7–12 years (from 0.6 to 3.5%) and in boys (from 1.3 to 3.6%) (Table 5).

### The Associations of Demographic, Geographic, Anthropometric Factors, and Obesity With Hypertension and Hypertension Phenotypes

Of the 15,143 records, 11,450 were with complete information on BP, weight, height, WC, HC, age, sex, setting and region. The demographic characteristics between the included (*n* = 15,143) group and excluded (*n* = 3,460) group were compared (Table 1). Of note, the included participants were relatively younger than the excluded individuals across all survey rounds (*P* < 0.05), except for in CHNS 2004 and 2011, where no sex difference was observed between the two groups (all *P* > 0.05).

Table 6 shows the associated demographic, geographic and anthropometric factors of hypertension and phenotypes. Age, region, BMI, WC, general obesity, and central obesity were found to be associated with hypertension (*P* < 0.001). Compared with children aged 7–12 years, those aged older than 13 years and above were more likely to suffer from prehypertension (OR = 2.08, 95%CI: 1.82–2.37, *P* < 0.001) and stage 1 hypertension (OR = 1.81, 95%CI: 1.61–2.03, *P* < 0.001). Girls (vs. boys) were less likely to be affected by prehypertension (OR = 0.83, 95%CI: 0.73–0.95, *P* = 0.005), whereas no sex difference was observed in the prevalence of stage 1 and 2 hypertension. BMI and WC were significantly correlated with the prevalence of different hypertension phenotypes (all ORs with 95%CI were >1 and *P* < 0.05, except for the SDH). For prehypertension, stage 1 hypertension and stage 2 hypertension, general obesity was a positive factor, with ORs of 2.00 (95%CI: 1.47–2.73, *P* < 0.001), 2.62 (95%CI: 2.03–3.38, *P* < 0.001), and 3.68 (95%CI: 1.82–7.44, *P* < 0.001), respectively. Central obesity was linked to both stages of hypertension additionally (OR = 1.43, 95%CI: 1.16–1.78, *P* = 0.001 and 2.76, 95%CI: 1.48–5.14, 0.001). For IDH, advanced age (OR = 2.02, 95%CI: 1.77–2.31, *P* < 0.001), general obesity (OR = 1.83, 95%CI: 1.36–2.47, *P* < 0.001) and central obesity (OR = 1.50, 95%CI: 1.19–1.90, *P* = 0.001) were positively associated with higher odds, whereas females, rural residents and south region were negative factors (ORs=0.86, 0.86 and 0.59, all *P* < 0.05). Similar to IDH, three out of these factors (13–17 years age group, general obesity and central obesity) were related to the increased prevalence of ISH (ORs = 1.57, 3.56, and 2.43, all *P* < 0.05). However, only general obesity contributed to the elevated prevalence of SDH, with an OR of 7.27 (95%CI: 4.57–11.59, *P* < 0.001).

**Table 6:** Multivariable odds ratios of demographic, geographic, and anthropometric factors and obesity for childhood hypertension and phenotypes.

**Characteristic**	**Prehypertension**		**Hypertension**		**Stage 1 hypertension**		**Stage 2 hypertension**		**ISH**		**IDH**		**SDH**	
	**OR (95%CI)**	***P***	**OR (95%CI)**	***P***	**OR (95%CI)**	***P***	**OR (95%CI)**	***P***	**OR (95%CI)**	***P***	**OR (95%CI)**	***P***	**OR (95%CI)**	***P***
**SURVEY YEAR**														
1993	1	Ref	1	Ref	1	Ref	1	Ref	1	Ref	1	Ref	1	Ref
1997	1.17 (0.91–1.52)	0.226	1.09 (0.86–1.37)	0.484	1.11 (0.88–1.41)	0.380	0.74 (0.30–1.83)	0.517	1.93 (0.94–3.96)	0.073	0.97 (0.74–1.26)	0.800	1.25 (0.72–2.17)	0.428
2000	1.23 (0.96–1.59)	0.106	1.17 (0.93–1.46)	0.188	1.20 (0.95–1.51)	0.133	0.75 (0.31–1.82)	0.530	1.78 (0.86–3.68)	0.118	1.08 (0.83–1.40)	0.556	1.30 (0.75–2.27)	0.348
2004	1.33 (1.01–1.75)	0.043	1.61 (1.27–2.04)	<0.001	1.65 (1.30–2.10)	<0.001	1.00 (0.39–2.54)	1.000	2.73 (1.30–5.71)	0.008	1.58 (1.22–2.06)	0.001	1.13 (0.61–2.12)	0.695
2006	1.18 (0.88–1.58)	0.276	0.89 (0.68–1.17)	0.413	0.95 (0.72–1.26)	0.708	0.21 (0.05–0.97)	0.045	1.35 (0.58–3.14)	0.489	0.85 (0.62–1.16)	0.311	0.79 (0.40–1.57)	0.499
2009	1.61 (1.21–2.16)	0.001	1.60 (1.24–2.07)	<0.001	1.60 (1.23–2.08)	0.001	1.57 (0.67–3.68)	0.296	2.03 (0.93–4.45)	0.076	1.60 (1.20–2.13)	0.001	1.31 (0.69–2.49)	0.410
2011	1.35 (1.02–1.78)	0.035	1.12 (0.87–1.44)	0.377	1.18 (0.92–1.53)	0.192	0.33 (0.11–1.05)	0.060	1.19 (0.54–2.62)	0.666	1.11 (0.84–1.47)	0.475	1.10 (0.60–2.00)	0.754
2015	2.14 (1.62–2.85)	<0.001	2.08 (1.61–2.67)	<0.001	2.09 (1.61–2.71)	<0.001	1.53 (0.65–3.59)	0.327	2.48 (1.17–5.25)	0.018	2.16 (1.63–2.86)	<0.001	1.37 (0.73–2.55)	0.324
**AGE GROUP**														
7–12 years	1	Ref	1	Ref	1	Ref	1	Ref	1	Ref	1	Ref	1	Ref
13–17 years	2.08 (1.82–2.37)	<0.001	1.76 (1.56–1.97)	<0.001	1.81 (1.61–2.03)	<0.001	1.02 (0.63–1.63)	0.939	1.57 (1.15–2.14)	0.004	2.02 (1.77–2.31)	<0.001	0.92 (0.69–1.22)	0.553
**SEX**														
Male	1	Ref	1	Ref	1	Ref	1	Ref	1	Ref	1	Ref	1	Ref
Female	0.83 (0.73–0.95)	0.005	0.92 (0.82–1.03)	0.160	0.91 (0.81–1.03)	0.126	1.08 (0.69–1.70)	0.743	1.19 (0.87–1.63)	0.279	0.86 (0.75–0.98)	0.025	1.01 (0.77–1.33)	0.927
**LOCATION**														
Urban	1	Ref	1	Ref	1	Ref	1	Ref	1	Ref	1	Ref	1	Ref
Rural	1.08 (0.94–1.25)	0.283	0.94 (0.83–1.07)	0.358	0.94 (0.83–1.07)	0.346	1.03 (0.63–1.67)	0.918	1.42 (0.99–2.04)	0.055	0.86 (0.75–0.99)	0.042	1.16 (0.86–1.58)	0.328
**REGION**														
North	1	Ref	1	Ref	1	Ref	1	Ref	1	Ref	1	Ref	1	Ref
South	0.94 (0.82–1.08)	0.401	0.65 (0.58–0.73)	<0.001	0.65 (0.57–0.73)	<0.001	0.70 (0.44–1.12)	0.134	1.10 (0.78–1.55)	0.576	0.59 (0.52–0.68)	<0.001	0.76 (0.58–1.02)	0.063
**BMI (kg/m**^**2**^**)**	1.06 (1.02–1.10)	0.003	1.10 (1.07–1.14)	<0.001	1.10 (1.07–1.14)	<0.001	1.04 (1.01–1.08)	0.014	1.05 (1.00–1.10)	0.044	1.08 (1.04–1.12)	<0.001	1.12 (1.01–1.24)	0.030
**WC (cm)**	1.02 (1.01–1.03)	<0.001	1.02 (1.01–1.03)	<0.001	1.02 (1.01–1.03)	<0.001	1.03 (1.01–1.06)	0.012	1.04 (1.02–1.06)	<0.001	1.02 (1.01–1.03)	<0.001	1.00 (0.98–1.02)	0.804
**GENERAL OBESITY**														
Normal	1	Ref	1	Ref	1	Ref	1	Ref	1	Ref	1	Ref	1	Ref
Overweight	1.26 (1.00–1.59)	0.051	1.66 (1.38–2.01)	<0.001	1.68 (1.39–2.04)	<0.001	1.25 (0.56–2.76)	0.584	1.48 (0.84–2.59)	0.172	1.54 (1.24–1.91)	<0.001	2.47 (1.62–3.77)	<0.001
Obesity	2.00 (1.47–2.73)	<0.001	2.69 (2.10–3.44)	<0.001	2.62 (2.03–3.38)	<0.001	3.68 (1.82–7.44)	<0.001	3.56 (2.03–6.22)	<0.001	1.83 (1.36–2.47)	<0.001	7.27 (4.57–11.59)	<0.001
**CENTRAL OBESITY**														
Normal	1	Ref	1	Ref	1	Ref	1	Ref	1	Ref	1	Ref	1	Ref
Central Obesity	1.18 (0.91–1.53)	0.203	1.49 (1.21–1.83)	<0.001	1.43 (1.16–1.78)	0.001	2.76 (1.48–5.14)	0.001	2.43 (1.48–3.99)	<0.001	1.50 (1.19–1.90)	0.001	1.08 (0.69–1.72)	0.728

^*^Values were odds ratio (95% confidence interval); Odds ratio for each variable was adjusted for all other variables in the table; Ref, reference; BMI, Body Mass Index; WC, Waist Circumference;The comparisons were between hypertension groups and normotensives; The multivariable generalized estimating equations (GEE) logistic regression adjusted all variables listed.

## Discussion

The current study describes the prevalence and associated factors of hypertension and phenotypes in Chinese children and adolescents during 1991–2015. It provides novel insights into several aspects of the national challenge of childhood hypertension in China. From 1991 to 2015, the age-standardized prevalence of hypertension and phenotypes in Chinese children and adolescents increased dramatically. Sex and age disparities were found in the prevalence of childhood hypertension and phenotypes. For adolescents aged 13–17 years, general obesity and central obesity were positively associated with hypertension, whereas the South region was a negatively related factor. For IDH, sex, age, location (urban or rural), region (north or south), BMI, WC, general obesity and central obesity were associated factors. Older age group, general obesity and central obesity were related to the increased prevalence of ISH, and only general obesity associate with the elevated prevalence of IDH.

It is well-known that hypertension in children is associated with long-term adverse health effects (7). However, few studies reported the trends and related factors of childhood hypertension phenotypes. To the best of our knowledge, this study is the first to report the secular trends in the prevalence of hypertension phenotypes in such a large sample of Chinese children over more than two decades. Based on the stringent sampling approach and standardized procedures for BP measurements in CHNS, the quality of our results was largely ensured. In line with a national survey in the United States, significant increasing temporal trends in childhood BP and the prevalence of hypertension (from 8.5 to 19.2%) were revealed in our study (29). However, such an upward secular trend was not universally observed in some previous studies due to the heterogeneity in demographic characteristics and socioeconomic status in different settings. For example, the Youth Heart Project in Northern Ireland has reported that the DBP has decreased by about ten mmHg during the past decade (30). In addition, a survey-based study in the United States showed that both mean SBP and DBP declined by 0.7 and 4.3 mmHg among 17-year-old adolescents from 1999–2002 to 2009–2012 (31). Liang et al. successfully revealed increasing trends in the prevalence of prehypertension and hypertension in Chinese children and adolescents from 1991 to 2004 (32). Compared with their study, we extended the study period from 1991–2004 to 1991–2015, additionally assessed the prevalence of hypertension according to severity (stage 1 hypertension and stage 2 hypertension) and phenotype (ISH, IDH, and SDH), and explored the effects of demographic, geographic factors, and obesity on hypertension.

IDH plays a critical role in childhood hypertension, and the prevalence was estimated to be 6.2–14.1% in this study, accounting for 67.3–78.3% of all phenotypes of hypertension. IDH in children and adolescents was defined as a DBP ≥ 95th percentile and an SBP < 95th percentile for each age, sex, and height subgroup (10). It has been discovered that DBP levels were regulated by the renin-angiotensin-aldosterone system (RAAs) and vasoactive substances (33). Disease and unhealthy lifestyles may affect the regulation of RAAs and lead to a rise in DBP values (34–36).

Our results showed that the prevalence of hypertension and its phenotypes by sex and age subgroups increased dramatically over the recent two decades. Compared with girls, boys were more vulnerable to prehypertension and IDH. The sex differences in hypertension prevalence have been generally recognized as a result of hormones occurring at puberty (37). It has been reported that estrogen was essential for the enhancement of endothelium-dependent vasodilation and regulation of smooth muscle cells (37, 38). Besides, unhealthy behaviors, such as alcohol intake, sedentary lifestyle and obesity were more common in boys than girls in China, which may contribute to a greater risk of hypertension (39, 40). In the current study, the prevalence of hypertension among teenagers aged 13–17 years (ranging from 11.8 to 22.1%) was higher than that in 7–12 years old children (ranging from 4.5 to 18.2%). According to clinical studies and animal experiments, the changes in vascular elasticity as children grow may partly explain the age disparities in the prevalence of hypertension among children (41, 42). Moreover, the prevalence of childhood hypertension differed in regions and locations. In comparison with urban children, rural children had a lower prevalence of IDH, which may be resulted from a better lifestyle and environment in rural settings, including more healthy eating patterns, more physical activities and fewer air pollutions (43, 44). Similarly, children living in South China were less likely to be affected by hypertension, stage 1 hypertension and IDH. It has been estimated that diary sodium intake in North China is higher than that in South China, which might have attributed to the differences across geographic regions (45).

It is widely acknowledged that obesity is one of the most important factors responsible for elevated BP in children and adolescents (46, 47). In accordance with a population-based study in Nigeria, where BMI and WC were revealed as risk factors for hypertension and prehypertension, we found that both general obesity and central obesity were associated factors of childhood hypertension (48). Previous studies also stated that the proportion of obese children suffering from hypertension was three times higher than that of non-obese children (49, 50). However, the mechanisms by which obesity directly influence BP is still unclear. It is reported that sympathicotonia and insulin resistance could regulate BP level through changing vascular reactivity and reduced-sodium excretion (51, 52). Besides, the accumulation of visceral adipose tissue triggers an immune response, resulting in an excessive amount of free fatty acids (FFA) (53). The high concentrated FFA in portal vein circulation inhibits the clearance of insulin and activates the production of angiotensinogen. In addition, the unbalanced angiotensinogen affects vasoconstriction and sodium reabsorption and, therefore, increases BP and develops hypertension (53).

### Limitations

Several limitations should be considered when interpreting the results of this study. First, the national representativeness of CHNS could not be fully ensured, despite the large sample size and a wide range of economic and demographic variations. Second, BP was measured three times on a one-time visit rather than on three different occasions. The prevalence of hypertension might be overestimated (54). Third, people that were included at each study interval were not necessarily the same people, so the change in blood pressure over time may be related to sampling in each round. Fourth, several confounding factors, such as family economic level, family parent education, children's dietary patterns, comorbidities and medications were not accounted for in our multivariable analyses due to the absence of relative information.

## Conclusion

In conclusion, BP levels and prevalence of hypertension and phenotypes increased dramatically in Chinese children and adolescents from 1991 to 2015. Regional discrepancy, demographic features, BMI, WC, and overweight/obesity status were associated factors of hypertension among youths. Our findings call for actions to identify and prevent childhood hypertension in China.

## Data Availability Statement

The datasets presented in this study can be found in online repositories. The names of the repository/repositories and accession number(s) can be found in the article/Supplementary Material.

## Author Contributions

PS designed the study. XY, QY, WX, and PS managed and analyzed the data. XY and QY prepared the first draft. All authors were involved in revising the paper and gave final approval of the submitted versions.

## Conflict of Interest

The authors declare that the research was conducted in the absence of any commercial or financial relationships that could be construed as a potential conflict of interest.
