# Supplementary material for: Trends in Prevalence of Hypertension and Hypertension Phenotypes Among Chinese Children and Adolescents Over Two Decades (1991–2015)
Source: Front Cardiovasc Med. 2021 May 11;8:627741. doi: 10.3389/fcvm.2021.627741 (PMC8144307; doi:10.3389/fcvm.2021.627741)
Supplement: Supplementary file 1 [file Data_Sheet_1.docx]

**Title: Trends in prevalence of hypertension and hypertension phenotypes in Chinese children and adolescents over two decades (1991-2015)**

**TABLE S1** Investigated provinces and in each wave of CHNS1989-2015

| **Wave year** | **Investigated provinces** |
| --- | --- |
| 1989 | Liaoning, Jiangsu, Shandong, Henan, Hubei, Hunan, Guangxi, Guizhou |
| 1991 | Liaoning, Jiangsu, Shandong, Henan, Hubei, Hunan, Guangxi, Guizhou |
| 1993 | Liaoning, Jiangsu, Shandong, Henan, Hubei, Hunan, Guangxi, Guizhou |
| 1997 | Heilongjiang, Jiangsu, Shandong, Henan, Hubei, Hunan, Guangxi, Guizhou |
| 2000 | Liaoning, Heilongjiang, Jiangsu, Shandong, Henan, Hubei, Hunan, Guangxi, Guizhou |
| 2004 | Liaoning, Heilongjiang, Jiangsu, Shandong, Henan, Hubei, Hunan, Guangxi, Guizhou |
| 2006 | Liaoning, Heilongjiang, Jiangsu, Shandong, Henan, Hubei, Hunan, Guangxi, Guizhou |
| 2009 | Liaoning, Heilongjiang, Jiangsu, Shandong, Henan, Hubei, Hunan, Guangxi, Guizhou |
| 2011 | Beijing, Liaoning, Heilongjiang, Shanghai, Jiangsu, Shandong, Henan, Hubei, Hunan, Guangxi, Guizhou, Chongqing |
| 2015 | Beijing, Liaoning, Heilongjiang, Shanghai, Jiangsu, Shandong, Henan, Hubei, Hunan, Guangxi, Guizhou, Chongqing |


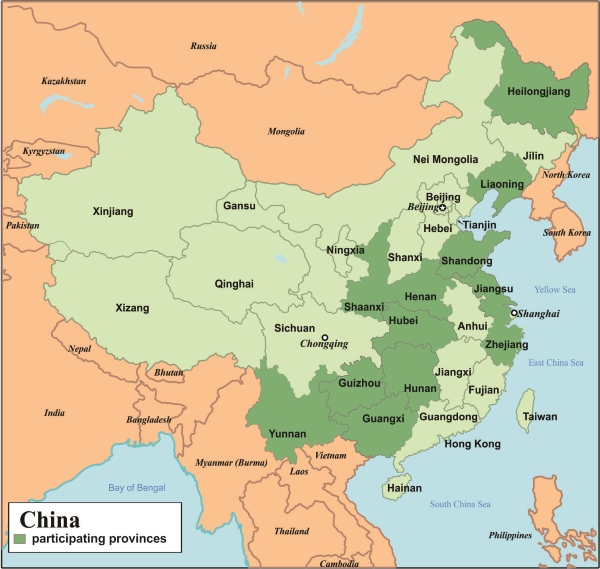


**FIGURE S1** Locations of investigated provinces in CHNS 1989-2015

*
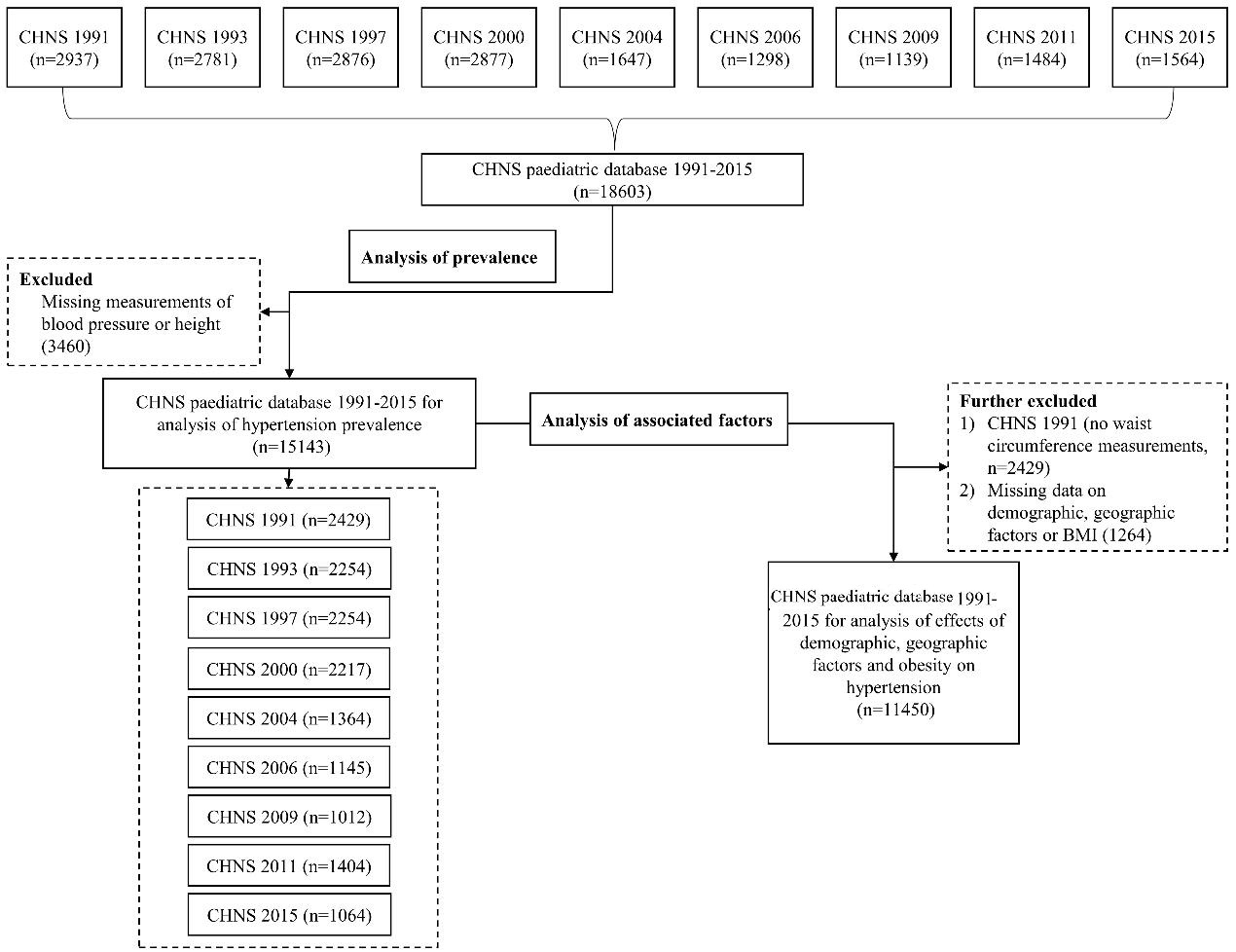
*

**FIGURE S2** Flowchart for selecting subjects in CHNS 1991-2015 for the analysis of childhood hypertension prevalence

*Note: the exclusion criteria were in an order as listed in the box, some subjects might meet several exclusion criteria simultaneously.
